# A national study to assess pharmacists’ preparedness against COVID-19 during its rapid rise period in Pakistan

**DOI:** 10.1371/journal.pone.0241467

**Published:** 2020-11-05

**Authors:** Iltaf Hussain, Abdul Majeed, Hamid Saeed, Furqan K. Hashmi, Imran Imran, Muqarrab Akbar, Muhammad O. Chaudhry, Muhammad Fawad Rasool

**Affiliations:** 1 Department of Pharmacy Practice, Faculty of Pharmacy, Bahauddin Zakariya University, Multan, Pakistan; 2 University College of Pharmacy, Allama Iqbal Campus, University of the Punjab, Lahore, Pakistan; 3 Department of Pharmacology, Faculty of Pharmacy, Bahauddin Zakariya University, Multan, Pakistan; 4 Department of Political Science, Bahauddin Zakariya University, Multan, Pakistan; 5 School of Economics, Bahauddin Zakariya University, Multan, Pakistan; Faculty of Science, Ain Shams University (ASU), EGYPT

## Abstract

To evaluate the pharmacist’s preparedness against the COVID-19 during its rapid rise period in Pakistan, an online cross-sectional study was carried out from March 30 to May 22, 2020 among the pharmacists using a pre-validated self-administered questionnaire. A total of 1149 participants completed the survey, amongst which 430(37.9%) were working as retail pharmacists, 216 (18.8%) as community pharmacists, and 213(18.5%) as hospital pharmacists. The mean COVID-19 knowledge score of the participants was 6.77±0.5, which indicated that 84% of them had good knowledge about COVID-19. The multiple linear regression model revealed that attitude was significantly associated with gender (p = 0.001), marital status (p<0.0001) and resident (p = 0.013). The mean practice score was 2.85±0.4, showing that 94% of the participants were following adequate preventive practices against this infection. The results from this study suggest that Pharmacists demonstrated good knowledge, positive attitudes, and acceptable practices regarding COVID-19.

## Introduction

The coronavirus disease 2019 (COVID-19) is the respiratory infection caused by novel coronavirus [1,2]. It was first reported in December 2019 from Wuhan, Hubei province, China, and spread rapidly to over 213 countries [3,4]. As of April 15, 2020, over 1.9 million confirmed cases of COVID-19 have been reported globally with a death toll of 123,126 patients (death ratio 6.4%) [5]. The US Center for Disease Control and Prevention (CDC) has recognized it as a serious public health threat and World Health Organization (WHO) declared it as a Public Health Emergency of international concern [5,6]. It is stated that severe acute respiratory syndrome coronavirus 2 (SARS- CoV2) is more contagious than the Middle East respiratory syndrome coronavirus (MERS-CoV) and SARS-CoV [7]. The primary route of COVID-19 transmission is airborne droplets or direct contact with an infected person and surface or object. The COVID-19 is a respiratory illness that is associated with symptoms like cough, fever, myalgia, and difficulty in breathing (in more severe cases) [3,4].

There is a rapid rise in the number of coronavirus cases in Pakistan. To date (June 15, 2020) 144,478 laboratory-confirmed cases of COVID-19 have been reported in Pakistan. To control the spread of COVID-19, the government of Pakistan took unrivaled steps including the suspension of public transportation, the closing of school, college, and universities, and isolation and care for infected and suspected cases. The government authorities had lockdown Pakistan until May 09, 2020, so that people can stay at home and may not be involved in the spreading of this infection [8].

During the current pandemic, it is recognized that community pharmacies may be the first point of contact for individuals with COVID-19 related health concerns [9]. The current situation highlighted the important actions that pharmacists can take as part of the global response to the pandemic. These include the provision of public health advice and education on personal and environmental hygiene, and making appropriate referrals in cases of suspected symptoms [10]. Pharmacist’s adherence to the control measures is very important to control and decrease the spread of COVID-19, which is greatly affected by knowledge, attitude, and practice (KAP) concerning COVID-19 [11,12]. There are a few published reports that were focused on assessing COVID-19 related knowledge of health care professionals in Pakistan [13–16]. To the best of our knowledge, there is no previous report of a nationwide study that has assessed the knowledge, attitude, and practices of pharmacists in Pakistan. The aim of the current study was to investigate the pharmacist’s knowledge, attitude, and practices regarding COVID-19 during the rapid rise period of the COVID-19 pandemic in Pakistan.

## Materials and methods

### Ethics statement

The study was approved by the ethical committee of the Department of Pharmacy Practice, Faculty of Pharmacy, Bahauddin Zakariya University, Multan Pakistan (reference number Acad/17/20/5).

### Study population

This cross-sectional web-based survey was conducted from March 30 to May 22, 2020. The study population consisted of pharmacists who were working in different professional fields in Pakistan, which include, academia, retail and community pharmacies, hospitals, and drug inspectors. An online questionnaire was designed using Google forms (Google LLC. USA) and its online link was shared through various social media platforms. The participants could view the question by simply clicking on the shared link and answer the questions. The first page of the questionnaire comprised of a short introduction regarding the objectives, procedures, declaration of confidentiality and anonymity, and the volunteer nature of the participants. Only pharmacists with a Pakistani Nationality were included in this study.

### Study instrument

An extensive literature search was carried out for developing and finalizing of the study questionnaire [3,7,17]. For evaluating the clarity of the questions, the developed questionnaire was first circulated amongst the senior practicing pharmacists. The suggested changes were incorporated into the questionnaire and then its reliability was tested by conducting a pilot study in 23 participants. A Cronbach alpha value of 0.63 was obtained from the pilot study, which showed that the developed instrument was reliable.

The questionnaire was designed in English and comprised of a series of questions portioning to socio-demographics, knowledge, attitude, and practice of pharmacists regarding COVID-19 as shown in Table 1. The knowledge part contains 7 questions (K1-K7) and was answered on true/false/I don’t know basis. A right answer was assigned 1 point and 0 point was assigned to incorrect/unknown answers. The total score for pharmacists varied between 0 (no correct answer) and 7 (all correct answer). The score ≥5 indicated good knowledge and <5 evaluated as poor knowledge. The third portion was related to attitude and responses were collected on a 3-point Likert scale. A mean score ≥2 indicated a positive attitude and ≤1 showed a negative attitude. The final part was regarding Practice toward COVID-19 and each correct response concerning the practice toward COVID-19 was given one point (rang: 0–3). The details regarding the study questionnaire and the informed consent form are available in the supporting information (S1 Table).

**Table 1:** Demographics characteristics of pharmacists (n = 1149).

Demographics		Mean	Standard Deviation
Age		28	5
		Frequency	Percentage
**Gender**	Male	808	70.3
	Female	341	29.7
**Marital status**	Married	675	58.7
	Un-married	474	41.3
**Educational level**	Bachelor	393	34.2
	Master	231	20.1
	M.Phil.	345	30.0
	Doctorate	180	15.7
**Residence**	Khyber pakhtunkhwa	445	38.7
	Punjab	325	28.3
	Sindh	164	14.3
	Balochistan	116	10.1
	Islamabad	99	8.6
**Occupation**	Retail pharmacist	330	28.7
	Community pharmacist	216	18.8
	Hospital pharmacist	213	18.5
	Teacher	179	15.6
	Drug inspector	211	18.4

### Data analysis

Statistical analysis was performed using the statistical package for social science (SPSS) version 25.0 (IBM Corp. Armonk, NY). Descriptive statistics were reported as frequency, percentage, and mean scores. *T*-test and one-way analysis of variance (ANOVA) was used to compare the demographics (independent variable) with knowledge, attitude, and practice score (dependent variable). Multivariable linear regression analysis using all of the demographic variables as independent variables and knowledge, attitude, and Practice score as the outcome variable was conducted to identify factors associated with knowledge, attitude, and practice. Pearson’s correlation test was applied to evaluate the relationship between knowledge and practice scores.

## Results

**Fig 1:**
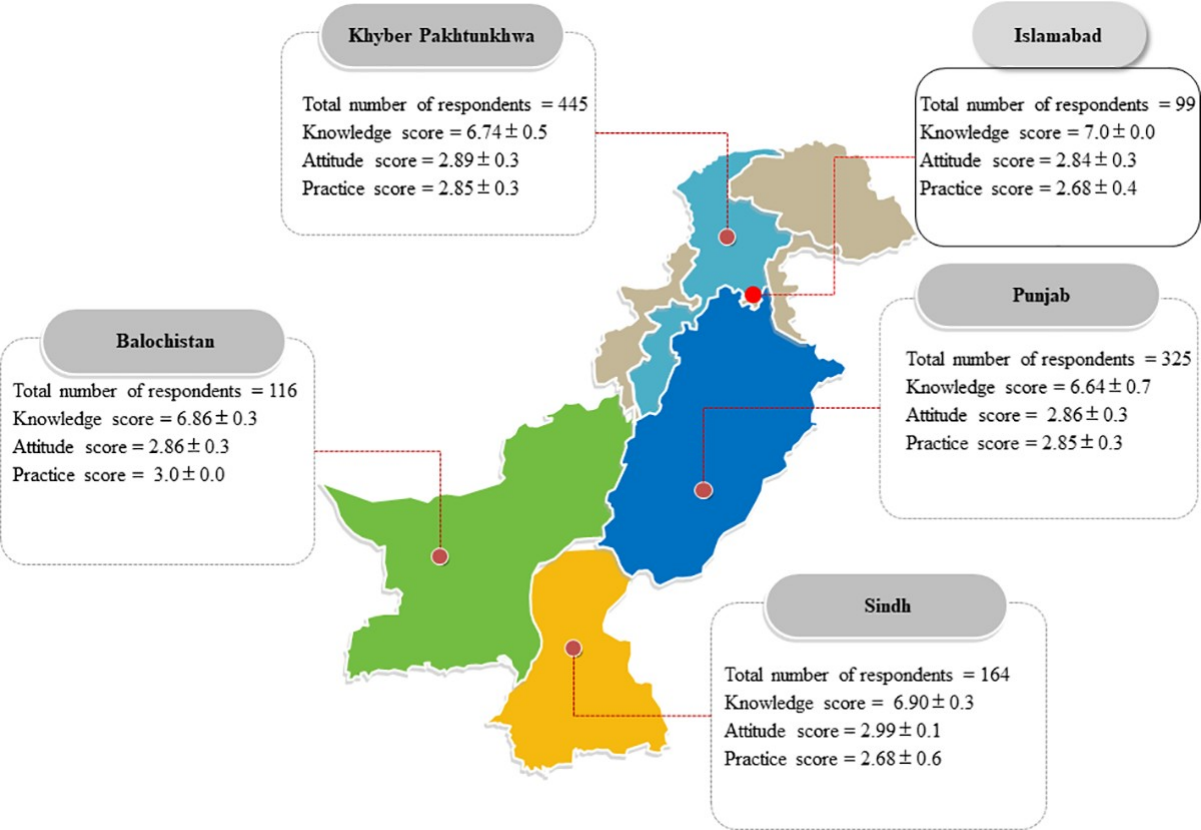
The geographic location of the participants of this study along with their knowledge, attitude, and practice scores.

A total of 1149 participants completed the survey, amongst which 808 (70.3%) were male and 341 (29.7%) were female. Also, 675 (58.7%) were married and 474 (41.3%) were unmarried. The mean age of participants was 28 years (standard deviation [SD]: 8.0, range: 20–45). Most of the participants were from Khyber Pakhtunkhwa 445(38.7%) followed by Punjab 325(28.3%), Sindh 164(14.3%), and Balochistan 116(10.1%). Most of the participants were working as retail pharmacists 430(37.9%), community pharmacists 216 (18.8%), and hospital pharmacists 213(18.5%). The demographic data related to the study participants can be seen in Table 1 and Fig 1.

The mean COVID-19 knowledge score was 6.77±0.5, which indicates that 84% of the participants had good knowledge about COVID-19 as shown in Table 2. The overall practice of the participants regarding COVID-19 prevention was satisfactory. The mean practice score was 2.85±0.4 which shows that 94% of the participants performed preventive practices to prevent infection transmission as shown in Table 2.

**Table 2:** Differences in pharmacist’s knowledge, attitude and practice scores regarding COVID-19 by demographic variables.

	Knowledge score		Attitude score		Practice score	
Age (mean±SD)	mean±SD	P-value	mean±SD	P-value	mean±SD	P-value
28.5±4.7	6.7±0.5	<0.0001	1.9±0.5	<0.0001	2.8±0.4	<0.0001
**Gender**						
Male	6.74±0.6	<0.0001	2.87±0.3	0.005	2.81±0.4	0.18
Female	6.86±0.3		2.93±0.2		2.86±0.3	
**Marital status**						
Married	6.80±0.5	0.02	2.95±0.2	<0.0001	2.75±0.4	<0.0001
Un-married	6.73±0.5		2.80±0.4		2.93±0.2	
**Education**						
Bachelor	6.67±0.5	<0.0001	2.87±0.3	0.27	2.87±0.3	<0.0001
Master	6.71±0.8		2.91±0.2		2.86±0.3	
M.Phil.	6.86±0.3		2.88±0.3		2.71±0.5	
Doctorate	6.91±0.2		2.91±0.2		2.91±0.2	
**Residence**						
Kpk	6.74±0.5	<0.0001	2.89±0.3	0.001	2.85±0.3	<0.0001
Punjab	6.64±0.7		2.86±0.3		2.85±0.3	
Sindh	6.90±0.3		2.99±0.1		2.68±0.6	
Balochistan	6.86±0.3		2.86±0.3		3.00±0.0	
Islamabad	7.00±0.0		2.84±0.3		2.68±0.4	
**Occupation**						
Retail pharmacist	6.70±0.7	<0.0001	2.90±0.3	<0.0001	2.85±0.3	<0.0001
Community pharmacist	6.70±0.6		2.76±0.4		2.77±0.5	
Hospital pharmacist	6.77±0.4		2.98±0.1		3.00±0.0	
Teacher	7.00±0.0		2.91±0.2		2.91±0.2	
Drug inspector	6.77±0.4		2.88±0.3		2.61±0.4	
**Total score**	6.77±0.5		2.88±0.3		2.85±0.4	

Data were expressed as mean±SD. t-test and ANOVA were used to a comparison between demographic characteristics of pharmacists and their knowledge, attitude, and practice scores. * P<0.05.

The multiple linear regression model revealed that the knowledge score was significantly associated with gender (β = 0.085, p = 0.21), education (β = 0.084, p<0.0001), resident (β = 0.06, p<0.0001) as shown in Table 3. The multiple linear regression model revealed that attitude was significantly associated with gender (β = 0.075, p = 0.001), marital status (β = -0.175, p<0.0001) and resident (β = -0.019, p = 0.013) as shown in Table 3. The multiple linear regression model revealed that the practices were significantly associated with gender (β = 0.086, p = 0.002), marital status (β = 0.179, p<0.0001), and occupation (β = -0.40, p<0.0001) as shown in Table 3.

**Table 3:** Association between demographics and the participant knowledge, attitude, and practice score using multiple linear regression analysis.

**Knowledge**	**Predictor**	**Coefficient**	**Standard Error**	***t-value***	***p*-value**
	(Constant)	6.270	.086	72.524	<0.0001
	Gender	.085	.037	2.312	0.021*
	Marital status	.006	.033	.188	0.851
	Educational level	.084	.015	5.533	<0.0001*
	Residence	.060	.013	4.771	<0.0001*
	Occupation	.021	.011	1.871	.062
**Attitude**	(Constant)	3.073	.054	57.047	<0.0001
	Gender	.075	.023	3.294	0.001*
	Marital status	-.157	.021	-7.556	<0.0001*
	Educational level	-.007	.009	-.708	0.479
	Residence	-.019	.008	-2.484	0.013*
	Occupation	-.001	.007	-.149	0.881
**Practices**	(Constant)	2.563	.067	38.533	<0.0001
	Gender	.086	.028	3.063	0.002*
	Marital status	.179	.026	6.957	<0.0001*
	Educational level	.015	.012	1.319	<0.188
	Residence	-.013	.010	-1.294	0.196
	Occupation	-.040	.009	-4.533	<0.0001*

* Statistically significant (p < 0.05).

Most of the survey participants were aware of the nature of infection (98.5%), signs and symptoms (98.5%), risk factors (98.6%), and management of the infection through early symptomatic and supportive treatment (98.5%). Most of the participants knew that COVID-19 spread can be prevented by wearing masks (95.6%). The prevalence of knowledge is shown in Table 4. The current study resulted that 93% of the participants were agreed that COVID-19 will be successfully controlled and 89% had confidence that Pakistan can win the battle against COVID-19. Around 86% disagreed with the statement that transmission of COVID-19 can be prevented by taking antibiotics. Attitude toward COVID-19 can be seen in Table 4. Most of the participants washing hands and faces with soap or sanitizer (94.2%), practicing social distance of at least one meter from the patient and other healthcare workers (95.7%), and wearing personal protective equipment during interaction with the patient including COVID-19 patients (93%). The practice-related results of pharmacists toward COVID-19 can be seen in Table 4.

**Table 4:** Knowledge, attitude and practices responses of pharmacist regarding COVID-19.

Knowledge related Questions (correct answer)			Correct Responses
COVID-19 is a viral infection (True)			1132(98.5)
The possible sign and symptoms of COVID-19 are fever, sore throat, cough, myalgia and shortness of breath (SOB) (True)			1132 (98.5)
COVID-19 is the same illness as flu or cold (True)			1021(88.9)
Currently, there is no effective treatment for COVID-19, but early symptomatic and supportive treatment can help most patients recover from the infection (True)			1132 (98.5)
People with a compromised immune system and old age people are at more risk of developing the infection (True)			1133 (98.6)
People in crowded places are at increased risk of getting affected by the disease.			1133 (98.6)
Wearing generally medical mask can prevent the spread up of infection by COVID-19 virus (True)			1098 (95.6)
**Attitude related Questions**			**Frequency (%)**
Do you agree that COVID-19 will finally be successfully controlled?		Agree	1069 (93.0)
		Neutral	32 (2.8)
		Disagree	48 (4.2)
Do you have confidence that Pakistan can win the battle against COVID-19 virus?		Agree	1033 (89.9)
		Neutral	34 (3.0)
		Disagree	82 (7.1)
Do you agree that transmission of COVID-19 infection can be prevented by taking antibiotics?		Agree	130 (11.3)
		Neutral	32 (2.8)
		Disagree	987 (85.9)
**Practice related questions**			**Frequency (%)**
I am using soap or sanitizer to wash hands and face.		Yes	1082 (94.2)
		No	67(5.8)
I avoid unnecessary close contact and practice social distancing and keep at least 1-meter distance from patients and other healthcare workers.		Yes	1100(95.7)
		No	49(4.3)
During interaction with the patient (including COVID-19 patient), I wear the necessary personal protective equipment such as masks, gloves, and gown, etc.		Yes	1068(93.0)
		No	81(7.0)

## Discussion

The current study was conducted among the pharmacist community to examine the KAP toward COVID-19. The results showed that participants have overall good knowledge, positive attitude, and acceptable preventive practices. The mean knowledge score was 6.77±0.5 which indicates that 84% of the participants had good knowledge about COVID-19. Most of the participants knew the nature of infection, possible signs and symptoms, and risk factors. The majority of the participant knew that there is no effective treatment for COVID-19 but supportive and symptomatic treatment helps in the recovery of the patients from COVID-19 and wearing masks can prevent the spread up of infection by COVID-19 virus. Most of the participants held a positive attitude towards COVID-19, as 93% of the participants agreed that this infection will be successfully controlled and 89.9% were confident that Pakistan will win this battle against COVID-19. Around 86% of the participants disagreed with the statement that COVID-19 transmission can be prevented by taking antibiotics. Most of the participants adopted good preventive practices by washing hands with soap or sanitizer (94.2%), keeping a social distance of one meter from patient and healthcare worker (95.7%), and wearing personal protective equipment during interaction with patients (93%).

The pharmacist knowledge and practice in complying precautionary measures can create awareness among patients and provide an important message to society [3]. The findings of the current study demonstrated that majority of pharmacist have good knowledge toward COVID-19 (84%), which was consistent with studies reported from Pakistan (93.2%), China (84%) [1], Iran (90%) [17], but higher than Jordon (40%) [7]. Similarly, a study reported from Iran claimed that 56.5% of respondents have sufficient knowledge regarding the transmission, symptoms, and treatment of COVID-19, this number was lower as compared to our study [18].

The attitude of the participants toward COVID-19 was optimistic, most of the pharmacist agreed that COVID-19 infection will finally be successfully controlled (93%) and Pakistan can win the battle against COVID-19 (89.9%) and 86% disagreed with the use of the antibiotic in the prevention of COVID-19 transmission. These findings were consistent with the study reported from China, where 97% of the participants had confidence that China could win the battle against COVID-19, and 91% of the participants agreed that the spread of this infection will be controlled successfully [3]. Similarly, higher than Peru, where 62% of the participants were confident that the government successfully controlled COVID-19 outbreak [19].

The good practices of pharmacists regarding COVID-19 prevention are imperative in effectively dealing with COVID-19 positive patients and can subside the associated risks [10]. Since, COVID-19 is now a pandemic, the pharmacist must follow and implement good hygienic conditions and adopt precautionary measures such as wearing gloves, protective clothing, goggles, face mask, and maintain suitable social distance from patients as well as from other healthcare workers [6,8,10]. The current study demonstrated that the majority of the pharmacist adopted good preventive practice such as washing hands with soap or sanitizer (94.2%), which was consistent with the study reported from China (89%) [3], Iran (86%) [17] and Peru (98.2%) [19]. In the present study, most of the participants adopted a social distance of one meter from the patient and healthcare worker (95.7%) and wearing personal protective equipment during interaction with patients (93%). This number was in line with studies reported from China, where 89% of the participants keeping social distance [1] but higher than Peru, where 59.9% of the participants practiced mask as a preventive measure [19].

## Limitations

In the present study, most of the respondents (pharmacists) were from urban areas and had easy access to the internet but it is pertinent to mention that a considerable number of pharmacists working in rural areas of Pakistan have very limited access to the internet and their knowledge, attitude, and practices (KAP’s) regarding COVID-19 were not evaluated in this study. This may be a limitation of this study. Moreover, the majority of the respondents were young pharmacists (mean age 28 years) who had access to the internet and their KAP’s cannot be compared with that of senior pharmacists, who are expected to have lower KAP scores due to limited access to information sources on the internet. Finally, the KAP’s of pharmacists can be affected by job-related stress especially in the current rapid rise period of COVID-19. Therefore, it will be more appropriate to discuss the level of stress experienced by the pharmacists during this pandemic along with their KAP scores.

## Conclusion

Our findings suggest that pharmacists in Pakistan have good knowledge, positive attitudes, and reasonable practices regarding COVID-19. To further ensure effective management of COVID-19, the knowledge and practices of the pharmacists should be continuously updated with the current up to date information. Therefore, the Government and the professional health associations should initiate focused trainings and seminars on various aspects related to diagnosis, management, and prevention of COVID-19.

## Supporting information

S1 Table The Questionnaire to evaluate the knowledge, attitudes, and practices of practicing pharmacists regarding the COVID-19 pandemic in Pakistan. (DOCX)
