# Supplementary material for: A national study to assess pharmacists’ preparedness against COVID-19 during its rapid rise period in Pakistan
Source: PLoS One. 2020 Nov 5;15(11):e0241467. doi: 10.1371/journal.pone.0241467 (PMC7644061; doi:10.1371/journal.pone.0241467)
Supplement: S1 Table — (DOCX) [file pone.0241467.s001.docx]

# Supporting information

The informed consent form and the study questionnaire used for assessing pharmacist’s knowledge, attitude, and practices regarding COVID-19 in Pakistan are given below.

# Informed consent form

Corona-virus disease (COVID-19) is a great threat to the world. This disease has spread to various countries including Pakistan. The study aims to assess the knowledge, attitude, and practices toward the COVID-19 in the practicing pharmacists of Pakistan.

This is an online cross-sectional study, the questionnaire comprises of questions regarding your demographic information, knowledge, attitudes, and practices about COVID-19. This questionnaire will hardly take your 5 minutes. All the information we obtain will remain strictly confidential and your answers and name will never be revealed. Besides, if you are not obliged to answer any question you do not want to, and you may stop at any time.

This study is not to evaluate or criticize you, so please do not feel pressured to give a specific response and do not feel shy if you do not know the answer to a question. We are not expecting you to give a specific answer; We would like you to answer questions honestly. Feel free to answer questions at your own pace.

Do you agree to participate in this survey?

Yes ___ No ___

# Questionnaire

**Table S1: The Questionnaire to evaluate the knowledge, attitudes, and practices of practicing pharmacists regarding the COVID-19 pandemic in Pakistan.**

| Questions | Responses |
| --- | --- |
| **K1.** COVID-19 is a viral infection  **K2.** The possible sign and symptoms of COVID-19 are fever, sore throat, cough, myalgia and shortness of breath (SOB)  **K3.** COVID-19 is the same illness as flu or cold  **K4.** Currently, there is no effective treatment for COVID-19, but early symptomatic and supportive treatment can help most patients recover from the infection  **K5.** People with a compromised immune system and old age people are at more risk of developing the infection  **K6.** People in crowded places are at increased risk of getting affected by the disease.  **K7.** Wearing generally medical mask can prevent the spread up of infection by COVID-19 virus  **A1.**Do you agree that COVID-19 will finally be successfully controlled?  **A2.** Do you have confidence that Pakistan can win the battle against the COVID-19 virus?  **A3.** Do you agree that transmission of COVID-19 infection can be prevented by taking antibiotics?  **P1.** I am using soap or sanitizer to wash hands and face.  **P2**. I avoid unnecessary close contact and practice social distancing and keep at least 1-meter distance from patients and other healthcare workers.  **P3**. During interaction with the patient (including COVID-19 patient), I wear the necessary personal protective equipment such as masks, gloves, and gown, etc. | True False I don’t know  True False I don’t know  True False I don’t know  True False I don’t know  True False I don’t know  True False I don’t know  True False I don’t know  Agree Neutral Disagree  Agree Neutral Disagree  Agree Neutral Disagree  Yes No  Yes No  Yes No |
